# Elevated retinal cGMP is not associated with elevated circulating cGMP levels in a canine model of retinitis pigmentosa

**DOI:** 10.1371/journal.pone.0279437

**Published:** 2022-12-30

**Authors:** Laurence M. Occelli, Kelian Sun, Paige A. Winkler, Brandy J. Morgan, Simon M. Petersen-Jones

**Affiliations:** Department of Small Animal Clinical Sciences, College of Veterinary Medicine, Michigan State University, East Lansing, Michigan, United States of America; University of Florida, UNITED STATES

## Abstract

**Purpose:**

To investigate whether raised levels of retinal cyclic guanosine monophosphate (cGMP) was reflected in plasma levels in *PDE6A*^*-/-*^ dogs.

**Materials and methods:**

Retina was collected from 2-month-old wildtype dogs (*PDE6A*^*+/+*^, N = 6), heterozygous dogs (*PDE6A*^*+/-*^, N = 4) and affected dogs (*PDE6A*^*-/-*^, N = 3) and plasma was collected from 2-month-old wildtype dogs (*PDE6A*^*+/+*^, N = 5), heterozygous dogs (*PDE6A*^*+/-*^, N = 5) and affected dogs (*PDE6A*^*-/-*^, N = 5). Retina and plasma samples were measured by ELISA.

**Results:**

cGMP levels in retinal samples of *PDE6A*^*-/-*^ dogs at 2 months of age were significantly elevated. There was no significant difference in plasma cGMP levels between wildtype and *PDE6A*^*-/-*^ or *PDE6A*^*+/-*^ puppies. However, the plasma cGMP levels of the *PDE6A*^*-/-*^ puppies were significantly lower than that of *PDE6A*^*+/-*^ puppies.

**Conclusion:**

cGMP levels in the plasma from *PDE6A*^*-/-*^ was not elevated when compared to control dogs. At the 2-month timepoint, cGMP plasma levels would not be a useful biomarker for disease.

## Introduction

Inherited retinal degenerations (IRDs) are a group of conditions showing genetic heterogeneity that are recognized in several species, including humans and dogs. Mutations in the gene homologues in humans and dogs often result in markedly similar phenotypes, making the dog an important model for the understanding of disease mechanisms and development of therapeutic approaches aimed for the treatment of human IRDs (for a review see references [1–3]).

Cyclic guanosine monophosphate (cGMP) is an important secondary messenger in phototransduction, which is a G-protein signaling pathway. In the rod photoreceptor light stimulation of the photopigment rhodopsin, which is a G-protein coupled receptor (GPCR) rod opsin combined with the chromophore 11-*cis*-retinal, causes a conformational change. The light-activated rhodopsin in turn activates the G-protein (transducin) which then activates a cGMP-phosphodiesterase (PDE6 in photoreceptors). The activated PDE6 hydrolyzes cGMP and the reduced level of cGMP leads to closure of cGMP-gated channels in the plasma membrane and hyperpolarization of the photoreceptor cell. Mutations in genes coding for proteins in the transduction cascade lead to IRDs in humans, dogs and mice (both with spontaneously occurring mutations and genetically engineered mutations). Accumulation of cGMP in the retinae of animal models with mutations that cause a failure in rod phototransduction has been demonstrated in both mouse and dog models [4–7].

There are several mouse IRD models in which altered cGMP levels have been associated with photoreceptor degeneration. The model studied in most detail is the *retinal degeneration 1* (*rd1*) mouse which has a null mutation in the *Pde6b* gene. In this model, loss of PDE6 function results in photoreceptor death associated with elevated photoreceptor cGMP levels starting at about 6 days after birth (when Pde6 activity normally begins) [4, 8]. Mouse models with mutations in the alpha Pde6 subunit (*Pde6a*) have also been studied. In one, a p.V685M *Pde6a* mutation results in decreased Pde6 activity and accumulation of cGMP in rods leading to cell death [6, 7]. Another mouse model with a p.D670G *Pde6a* mutation has reduced Pde6 activity and a very slight increase in cGMP levels as shown by immunohistochemistry in postnatal day 11 old mice [7] but a reduction of cGMP levels by postnatal days 14–21 when assayed using a cGMP kit [6]. An additional mouse model with a p.R562W *Pde6a* mutation also has reduced PDE6 activity resulting in accumulation of cGMP. Mutations in other IRD genes have also been associated with cGMP accumulation including *Aipl1*, *Cngb1*, *Gucy2d*, *Prph2* and *Rho* [9].

Two dog models in which a failure of cyclic GMP-phosphodiesterase activity is reported include those with mutations in *PDE6B* and *PDE6A* [5, 10]. The *PDE6A* mutant dog has a rapid photoreceptor degeneration resulting from a 1 bp deletion in the *PDE6A* gene that was identified in the Cardigan Welsh corgi breed. The resulting frameshift causes a premature stop codon and a lack of the PDE6A protein (c.1939delA p.Asn616ThrfsTer39) [10, 11].

Cyclic GMP levels in human subjects with IRDs have been investigated. A recent paper from Kjellström et al reported that circulating cGMP levels were significantly elevated in members of a consanguineous family with RP due to a splice site mutation in *PDE6A* (IVS6+1G>A). The authors suggested that plasma cGMP had potential to be a biomarker for RP resulting from mutations in pathways predicted to lead to accumulation of retinal cGMP [12]. The authors considered that the loss of PDE6 function could result in increased extracellular retinal cGMP that “finds its way into the circulation”. They went onto suggest the use of animal models to test their hypothesis. The use of the level of circulating cGMP as a biomarker for IRDs may be problematic because elevation can be associated with a number of conditions such as cardiovascular disease; including higher blood pressure and cardiac failure as well as from elevated circulating natriuretic peptide [13]. Having a dog colony with a spontaneous functional *null* mutation in *PDE6A* gave us the opportunity to investigate in a controlled setting plasma cGMP levels in animals with elevated retinal cGMP levels and compare them to age-matched related control animals housed under identical conditions. In this study we showed *PDE6A* mutant animals had significantly elevated retinal cGMP. However this was not reflected in the systemic circulation suggesting there was not a detectable spillover of retinal cGMP into the systemic circulation and suggesting that plasma cGMP may not be a reliable biomarker for IRDs.

## Materials and methods

### Animals

The animals used were from dog colonies maintained at Michigan State University. They were housed under similar conditions exposed to 12hr light:dark cycles and fed a commercial dog diet. All experimental procedures were approved by the Michigan State University Institutional Animal Use and Care Committee (AUF number 05/14-090-00) and followed the Association for Research in Vision and Ophthalmology statement for the use of animals in ophthalmic and vision research.

### Sample collection

Retinal samples were collected from 2-month-old wildtype crossbred dogs (*PDE6A*^*+/+*^, N = 6, 2F, 2M, 2 sex unrecorded), that were being euthanized for studies unrelated to the current study, heterozygous dogs (*PDE6A*^*+/-*^, N = 4, 3F, 1M) and affected dogs (*PDE6A*^*-/-*^, N = 3, 2F, 1M) from a colony of Corgi cross beagle *PDE6A* mutant dogs. Following humane euthanasia by pentobarbitone overdose, retinae for cGMP assay were dissected and immediately flash frozen in liquid nitrogen and stored at -80°C until cGMP measurement. Eyes for immunohistochemistry were fixed in paraformaldehyde and processed as previously described [14]. Other body parts from euthanized dogs were supplied to other researchers.

Blood was collected between 12:00pm and 2:00pm to avoid circadian rhythm differences, from 2-month-old wildtype crossbred dogs (*PDE6A*^*+/+*^, N = 5, 1F, 4M), heterozygous dogs (*PDE6A*^*+/-*^, N = 5, 3F, 2M) and affected dogs (*PDE6A*^*-/-*^, N = 5, 3F, 2M). Plasma was treated with 5mM EDTA to inhibit endogenous phosphodiesterase activity. The samples were stored at -80°C until cGMP measurement.

### Cyclic GMP measurement

Retina samples were homogenized in 1 ml 0.1 M HCl by gentalMACS Dissociator (Miltenyi Biotec, Bergisch Gladbach, Germany) using the manufacturer’s protocol (protein_01) and centrifuged at 700 g for 10 minutes at 4°C to pellet solid material. The solution was transferred to a 1.5ml Eppendorf tune and centrifuged at 13,000g for 10 minutes at 4°C. Then 250 μl of the supernatant was transferred into a 10KD Spin column and centrifuged at 10,000g for 15 minutes or until 100 to 150μl solution passed through the column. This flowthrough from the spin column was used for the cGMP assay.

On the day of the cGMP assay, plasma was thawed on ice, centrifuged at 13,000g for 5 minutes and the supernatant was transferred to a new tube. Two hundred microliters (μl) were transferred into a 10KD Spin column (ab93349, Abcam, Cambridge, MA) and centrifuged at 10,000g for 15 minutes or until around 100 to 150 μl solution passed through the column. This flow through from the spin column was used for the cGMP assay.

The measurement of cGMP was carried out by the using the cGMP Complete ELISA kit (ab133052, Abcam, Cambridge, MA), following the manufacturer’s instructions for the kit, with samples being acetylated before measuring. The measurement was carried out at O.D. 405 nm with a Synergy H1 microplate reader (Biotek, Winooski, VT). The cGMP levels of the retina and plasma samples were calculated utilizing a five-parameter log-logistic fitted standard curve (via R) made from pure cGMP provided in the kit [15]. The cGMP levels were normalized to total protein content.

### Statistical analysis

Statistical analysis of cGMP levels was performed using Sigmaplot v14.5 (Systat Software, San Jose, CA). Data were checked for normality (Shapiro-Wilk test). A one way ANOVA was performed and pairwise comparisons made (Holm-Sidak method). Significance was set at p<0.05.

### Immunohistochemistry

Immunohistochemistry (IHC) was conducted on retinal sections from 2-month-old *PDE6A*^*+/-*^ and *PDE6A*^*-/-*^ dogs as previously described [14]. Sections were labeled using RPE65 antibody (mouse, 1:500; gift from Debra Thompson, Kellogg Eye Center, University of Michigan Medical School, Ann Arbor, MI) with Alexa Fluor donkey anti-mouse 488 secondary antibody (1:500) and cGMP (sheep, 1:3000; gift from Dr. HWM Steinbusch, Maastricht University Medical Center, Maastricht NL) with Alexa Fluor donkey anti-goat 568 secondary antibody (1:500) and nuclei were stained with 4′,6-diamidino-2-phenylindole (DAPI).

## Results

### Retinal cGMP measurements

**Fig 1:**
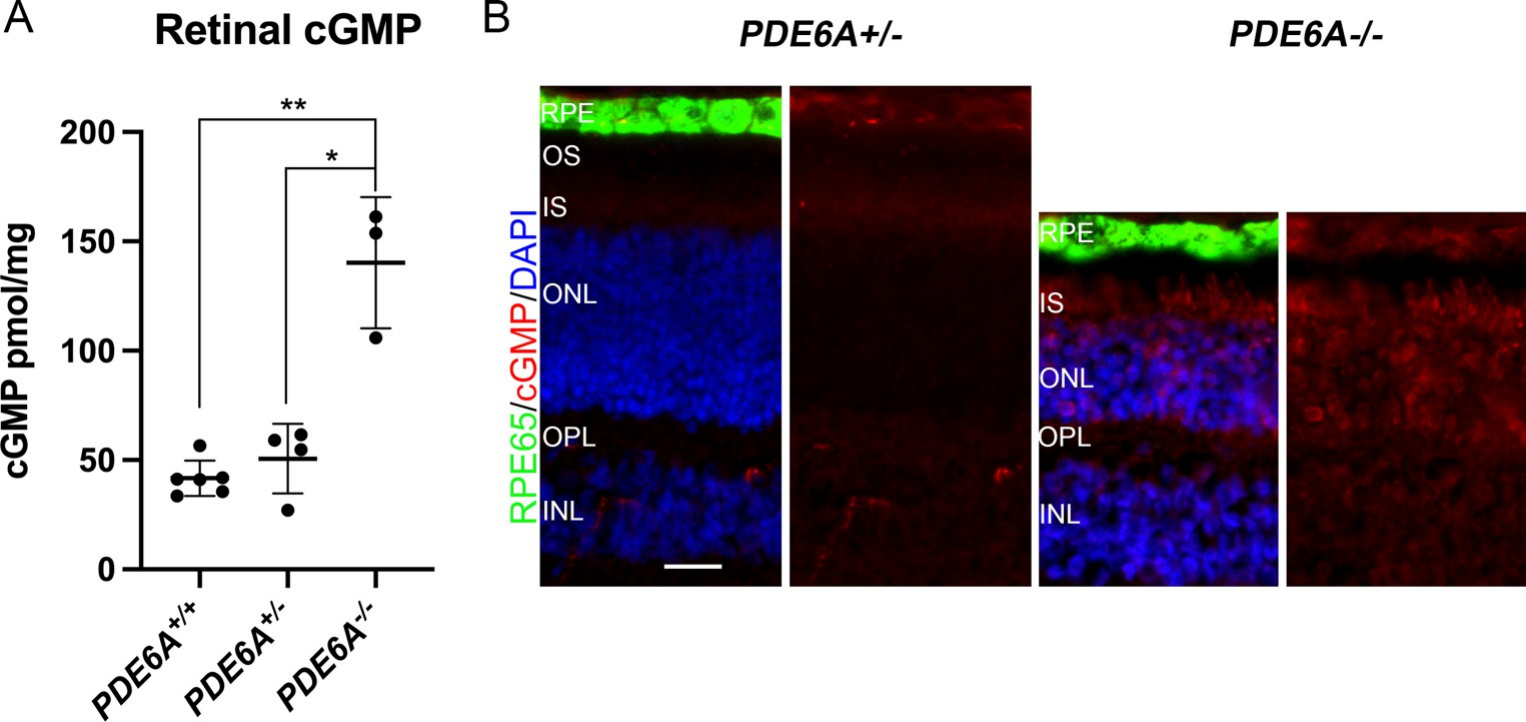
Retinal cGMP levels in 2-month-old animals. (A) Retinal cGMP is significantly elevated in *PDE6A*^*-/-*^ dogs compared to *PDE6A*^*+/+*^ and *PDE6A*^*+/-*^ dogs. (B) Retinal sections from *PDE6A*^*+/-*^ and *PDE6A*^*-/-*^ dogs showing cGMP (red) accumulation in the photoreceptors extending to include the cell bodies and synaptic terminals. For each genotype the left image shows cGMP (red), RPE cells (green) and DAPI nuclear counterstain (blue) and right image is of the same image showing cGMP (red) only, size bar 20 μm. RPE—retinal pigment epithelium; OS—photoreceptor outer segments; IS—photoreceptor inner segments; OPL—outer plexiform layer; INL-inner nuclear layer.

*PDE6A*^*-/-*^ animals had a mean retinal cGMP level of 181.44 ± 34.40 pmol cGMP/mg protein, while the *PDE6A*^*+/-*^ and the *PDE6A*^*+/+*^ animals had a mean value of 56.40 ± 5.03 and 44.81 ± 8.18 pmol cGMP/mg protein, respectively (Fig 1A). The retinal cGMP levels were significantly different between the groups F(2,10) = 36.43. p<0.0001. Pairwise comparisons showed that there was no difference between the retinal cGMP levels of *PDE6A*^*+/+*^ compared to *PDE6A*^*+/-*^ dogs (P = 0.268). The retinal cGMP levels of *PDE6A*^*-/-*^ dogs were significantly greater than that of both *PDE6A*^*+/+*^ (p<0.0001) and *PDE6A*^*+/-*^ dogs (p = 0.0035).

IHC on retinal sections from *PDE6A*^*+/-*^ and *PDE6A*^*-/-*^ dogs show accumulation of cGMP throughout the rod photoreceptors (Fig 1B).

### Plasma cGMP measurements

**Fig 2:**
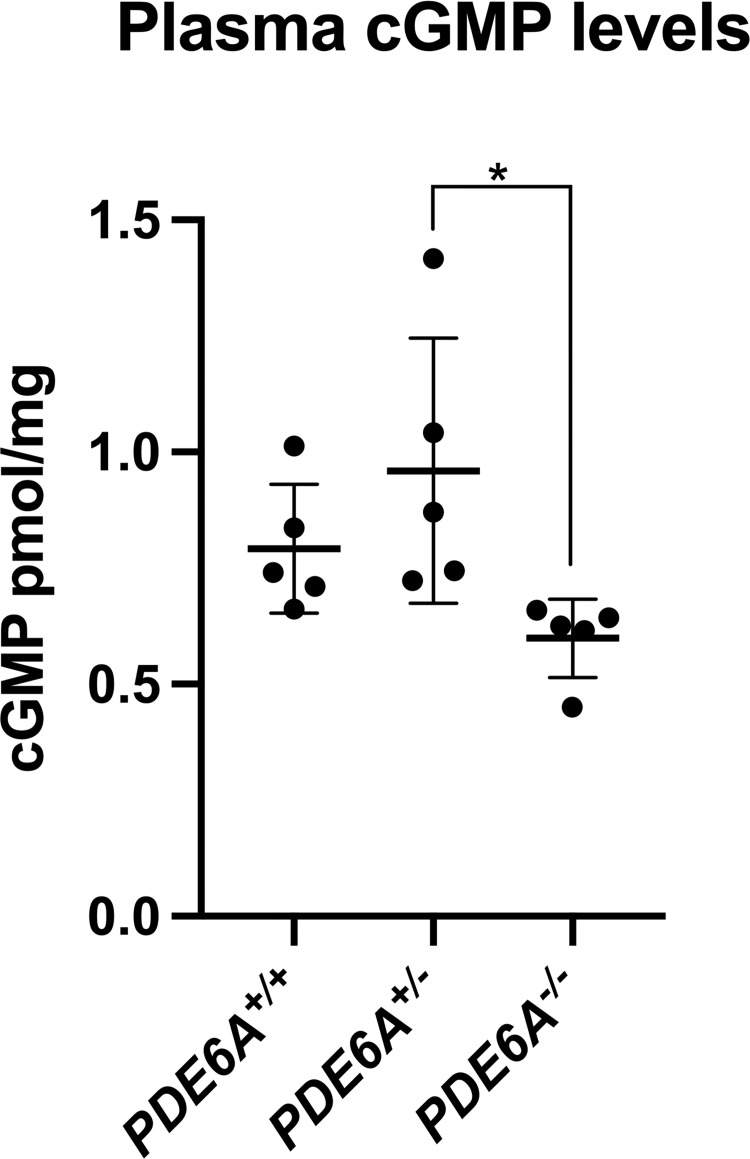
Mean plasma cGMP levels in 2-month-old animals. The mean serum cGMP levels of the *PDE6A*^*-/-*^ animals was lower than that the other two groups. The difference was only significant between *PDE6A*^*-/-*^ and *PDE6A*^*+/-*^ animals. * p = 0.032.

Mean plasma cGMP levels were: *PDE6A*^*-/-*^ 0.599 ± 0.085, *PDE6A*^*+/-*^ 0.960 ± 0.286 and *PDE6A*^*+/+*^ 0.792 ± 0.139, pmol cGMP/mg protein (Fig 2). The plasma cGMP levels were different between the groups F(2,12) = 4.525, p<0.034. The cGMP level in the *PDE6A*^*-/-*^dogs was significantly lower than for the *PDE6A*^*+/-*^ dogs (p = 0.032). There was no difference between *PDE6A*^*-/-*^ and *PDE6A* ^*+/+*^ dogs (p = 0.248) nor between *PDE6A*^*+/-*^ and *PDE6A* ^*+/+*^ dogs (p = 0.189).

Individual dog cGMP measurements are included in Supplementary information.

## Discussion

The main findings of this study were that *PDE6A*^*-/-*^ puppies had significantly elevated retinal cGMP levels at 2 months of age and this was due to accumulation of cGMP in the photoreceptor layer. The elevated retinal cGMP was not, however, reflected in circulating plasma cGMP levels. The mean plasma cGMP level of the *PDE6A*^*-/-*^ puppies was significantly lower that of the *PDE6A*^*+/-*^ animals but not significantly different from the wild-type controls. These findings in the dog are not in support of the hypothesis put forth by Kjellström et al who found human subjects with RP due to a *PDE6A* mutation had plasma cGMP levels twice that of controls. They hypothesized that the cGMP elevation could have resulted from “spill over” of elevated cGMP in the retina into the blood stream [12] a hypothesis supported by experiments showing that cGMP can be released in culture medium in animal model retinas [16]. They suggested that assaying plasma cGMP could be included as part of the investigation of suspected RP patients. The family in the Kjellström et al study had a mean age of 43 ± 16 years and had advanced retinal degeneration as assessed by optical coherence tomography. This would suggest that there was a reduced population of surviving rods to generate and accumulate cGMP seeming to make the “spill over” theory less likely. We used a dog model with a functional null mutation in PDE6A that ablates PDE6 activity [10]. The affected dogs accumulate cGMP in rod photoreceptors and this accumulation can be reversed by gene augmentation therapy to introduce a normal copy of *PDE6A* and restore rod phototransduction [17]. In the current study the levels of cGMP were measured at an age where the normal canine retina has reached maturity and the *PDE6A*^*-/-*^ retina still has a substantial population of rods with elevated cGMP levels as shown by cGMP assay and IHC (see Fig 1). Our previous detailed characterization of the *PDE6A*^*-/-*^ dog phenotype showed that in the central retina the number of rod nuclei per 100μm of retinal length was decreased from about 240 nuclei in control retina to 125 nuclei in the affected dogs [10] A potential limitation of the Kjellström et al study was that the RP patients were reported to be of Iraqi origin while the controls were healthy (presumably local) volunteers. The study was performed in Sweden and there was no mention of trying to match the ethnicity of the control subjects with that of the RP family possibly introducing differences due to background genetics or possibly environmental differences associated with different cultures. In our study we used littermate, age-matched phenotypically normal dogs heterozygous for the *PDE6A* mutation as well as colony sourced age-matched wild-type dogs. This was intended to minimize the potential effects of background genetics and environmental influences on circulating cGMP levels. Samples were also collected at the same time of day to remove the effect of any potential circadian differences in cGMP levels which in humans results in over 2.5x variation in plasma levels [18]. We cannot rule out that species differences might account for the difference in our findings to those reported by Kjellström et al. Indeed, previous studies have shown there can be a difference in blood PDE activity between species [19] although a direct comparison between dog and human does not appear to have been reported. When the similarity in eye size between dog and human is considered and yet the difference in body size between the 2 month-old puppies (weigh about 3 to 4 kg) compared to adult humans it seems that we would be more likely to see retinal spill over in the dog than in the human [20, 21]. Unexpectedly, the *PDE6A*^*-/-*^ puppies had a significantly lower plasma cGMP level than the heterozygotes. The animals used in this study for plasma cGMP assay were born within 2 days of each other were quite closely related and were all housed in the same facility. The *PDE6A*^*+/-*^ animals were all from a single litter and the *PDE6A*^*-/-*^ puppies from 2 litters. It is conceivable that because the *PDE6A*^*+/-*^ animals were all from the same litter that background genetics might be responsible for the difference between the plasma cGMP levels of *PDE6A*^*-/-*^ and *PDE6A*^*+/-*^ animals, rather than it being due to disease status. A much larger study would be required to see if the difference in plasma cGMP levels between *PDE6A*^*+/-*^ and *PDE6A*^*-/-*^ animals is a consistent finding. In addition, as we only tested animals at a single relatively early disease stage, future studies could be undertaken to see how the plasma cGMP levels change with age and retinal degeneration stage. We would expect the retinal cGMP levels in the *PDE6A*^*-/-*^ animals would decrease in parallel with the progressive loss of rod photoreceptors that occurs in this condition [10].

Martínez-Fernàndez de la Càmara et al investigated antioxidant-oxidant levels in peripheral blood of RP and Ushers syndrome patients and showed that they had elevated levels of plasma cGMP (as well as other antioxidant-oxidant markers) [22]. The causal mutations in these patients was not reported, so it is not possible to speculate which patients would be expected to have elevated retinal cGMP levels. This paper noted that age (and therefore stage of disease) had a statistically significant impact on peripheral blood antioxidant-oxidant markers (including cGMP). They postulated that the higher cGMP levels in RP patients were due to increased activation of soluble guanylate cyclase by nitrous oxide (NO) or carbon monoxide (CO). NO is upregulated due to oxidative stress from the retinal disease while increased CO could be a result of increased expression of heme oxygenase enzymes (HO-I and HO-II) in response to oxidative stress from the retinal disease [22]. The *PDE6A*^*-/-*^ dogs did have established rod photoreceptor loss at the age tested and in a previous study we found that there was peak levels of apoptosis at this age [10]. However, we cannot rule out that plasma cGMP might become elevated at more advanced disease stages associated with further activation of oxidative stress pathways.

## Conclusion

We found no evidence in the *PDE6A* dog model to support elevation of plasma cGMP levels as a biomarker for retinal degeneration associated with elevation of retinal cGMP levels.

## Supporting information

S1 Table cGMP levels for retinal and plasma samples. (XLSX)
